# Effectiveness of Self-Guided Virtual Reality–Based Cognitive Behavioral Therapy for Panic Disorder: Randomized Controlled Trial

**DOI:** 10.2196/30590

**Published:** 2021-11-22

**Authors:** Bokyoung Shin, Jooyoung Oh, Byung-Hoon Kim, Hesun Erin Kim, Hyunji Kim, Suji Kim, Jae-Jin Kim

**Affiliations:** 1 Institute of Behavioral Sciences in Medicine Yonsei University College of Medicine Seoul Republic of Korea; 2 Department of Psychiatry, Yonsei University College of Medicine Gangnam Severance Hospital, Yonsei University Health System Seoul Republic of Korea

**Keywords:** virtual reality, panic disorder, cognitive behavioral therapy, exposure therapy, intervention

## Abstract

**Background:**

Virtual reality (VR) is as effective a technique as traditional cognitive behavioral therapy (CBT) and a promising tool for treating panic disorder symptoms because VR exposure can be safer and has better acceptability than in vivo exposure and is more immersive than exposure through imagination. CBT techniques can be delivered more effectively using VR as well. So far, VR has required high-quality devices, but the development of mobile VR technology has improved user availability. At the same time, a well-structured form of VR can be reproduced and used anywhere. This means that VR can be used to provide a self-guided form of treatment and address the high treatment costs of evidence-based therapy and the lack of professional therapists. This study aimed to investigate the potential of self-guided VR as an alternative to high-cost treatment.

**Objective:**

The main goal of this study was to offer data about the efficacy of a mobile app-based self-led VR CBT in the treatment of panic disorder.

**Methods:**

A total of 54 subjects with panic disorder were enrolled in this study and randomly assigned to either the VR treatment group or waitlist group. The VR treatment was designed to be total 12 sessions for 4 weeks. The VR treatment consists of 4 steps in which patients are gradually exposed to phobic stimuli while learning to cope with panic symptoms in each stage. The effectiveness of treatment was assessed through the Panic Disorder Severity Scale, Hamilton Rating Scale for Depression, Body Sensations Questionnaire, Albany Panic and Phobia Questionnaire, Anxiety Sensitivity Index, State-Trait Anxiety Inventory, Hospital Anxiety and Depression Scale, Korean Inventory of Social Avoidance and Distress Scale, Korean Inventory of Depressive Symptomatology, and Perceived Stress Scale. In addition, physiological changes using heart rate variability were evaluated.

**Results:**

In within-group analyses, the VR treatment group exhibited improvements in panic disorder symptoms, anxiety, and depression after 4 weeks, while the waitlist group did not show any significant improvement. Compared to the waitlist group, the VR treatment group showed significantly greater improvements in the Panic Disorder Severity Scale in both completer analysis and intention-to-treat analysis. Heart rate variability in the VR treatment group showed improvement in normalized high frequency from baseline to postassessment with no significant differences in any outcome measure between groups.

**Conclusions:**

The self-guided, mobile app-based VR intervention was effective in the treatment of panic symptoms and restoring the autonomic nervous system demonstrating the validity of the use of VR for self-guided treatment. VR treatment can be a cost-effective therapeutic approach.

**Trial Registration:**

ClinicalTrials.gov NCT04985019; https://clinicaltrials.gov/ct2/show/NCT04985019

## Introduction

Panic disorder [1], with or without agoraphobia, is one of the most common mental disorders in the general population [2]. Panic disorder is characterized by a sudden anxiety with physiological symptoms including palpitations, sweating, and choking sensations accompanied by cognitive symptoms such as catastrophizing and fear of dying, which can lead to avoidance of particular places or situations [3]. Avoiding public places to reduce fear or panic prompt negative consequences that decrease the quality of life of patients [4-7].

As demonstrated in several studies, the most empirically supported psychosocial treatment for panic disorder is cognitive behavioral therapy (CBT) [8-10]. The classic form of CBT for panic disorder, proposed by Clark and Wells [11], includes education on dysfunctional thoughts, exposure to the feared situation, interoceptive exposure, cognitive restructuring, breathing retraining, and applied relaxation. These cognitive and behavioral techniques are designed to help patients realize and correct dysfunctional thoughts and behavior related to their anxiety [12].

The core contents of CBT for panic disorder are repeated exposure to feared situations and sensations, supported by coping skill trainings including breathing training and progressive muscle relaxation [9,13]. Previously, exposure therapy has been performed by imagination or in vivo exposure [14]. However, imagination by itself may not make patients fully immersed [15]. In vivo exposure has the disadvantages of temporal/spatial constraints and high costs. Patients with severe symptoms also tend to misrecognize and avoid in vivo exposure as aversive [16-18]. As an alternative, virtual reality (VR) exposure can be safer with better acceptability than in vivo exposure and is more immersive than exposure through imagination [19]. VR not only makes it easy to reach diverse places, such as an airplane, but it also has the advantage of the participant being able to easily escape from difficult situations during treatment by taking off the head-mounted display. Delivering other CBT techniques like abdominal breathing and progressive muscle relaxation with VR can increase effectiveness. Since VR can be used repeatedly, psychoeducation about the panic disorder such as prevalence, prognosis, possible effect, and side effect of each type of medication is possible without any additional manpower by using a virtual therapist [20].

As already noted, breathing training and progressive muscle relaxation are important parts of CBT for panic disorder as coping skill training with the purpose of modifying pathological breathing, training abdominal breathing [21,22], reducing general tension, and decreasing the risk of panic [23-25]. VR has advantages not only in exposure but also in coping skill training. For instance, in VR, if a virtual therapist demonstrates and teaches breathing and progressive muscle relaxation training, patients will be able to understand it more easily. There is evidence that the additive effects of breathing training plus exposure yielded better outcomes than exposure without breathing training [25]. In addition, progressive muscle relaxation in particular reduces panic symptoms when used in combination with interceptive exposure [26]. Accordingly, this simultaneous use of coping skill training and exposure technique can be thought of as an effective way to manage the panic symptoms. Since VR seems to lend an advantage to both coping skill training and exposure treatment, it will be of great help in simultaneous use of them as well.

Increasing numbers of studies have found VR is as effective a technique as traditional CBT and a promising tool for treating panic disorder symptoms [18,20,27-29]. Many studies have reported that VR and in vivo exposure show the same therapeutic effects, both being significantly better than the control group [18,20,27-32]. VR-based CBT could significantly reduce the number of panic attacks, level of depression, state and trait anxiety, and agoraphobia symptoms [28]. Even though these studies were conducted on a relatively small sample size, the consistent results support the use of VR as an effective tool in the treatment of panic disorder [33].

Recently, the development of mobile VR technology with low-cost devices have improved user availability [34]. So far, VR has usually been implemented as a therapist-led intervention, which made the VR costly. Contrary to this, self-guided VR has potential as an alternative to high-cost treatment [35,36]. Previously, some studies have explored the feasibility of a self-help VR app [20,37-39]. Lindner et al [20] demonstrated that the smartphone version of self-led VR exposure was as effective for treating public speaking anxiety within a therapist-led treatment format. Although effectiveness of self-led VR exposure was shown, VR was only a tool for exposure, indicating that it was partially self-led therapy [20]. Donker et al [37] implemented a fully self-guided VR-based CBT app for acrophobia, and it could significantly reduce acrophobia symptoms compared with the control group. However, this study relied on only self-reported measurement, so the treatment effect was not sufficiently validated by objective evaluation. More importantly, there was still no study that verified the effectiveness of self-guided VR for the treatment of panic disorder.

In this study, we developed a mobile app-based self-led VR CBT that included comprehensive components of CBT and various VR exposure contents. Patients learned the CBT techniques from a virtual therapist and were repeatedly exposed to realistic virtual environments. We studied whether this VR app for a self-help treatment was efficacious for panic disorder. It was hypothesized that individuals using a VR app would show greater changes in posttreatment measurement scores than the waitlist group. The effectiveness was assessed by the trained psychologists and through self-reported measurements. In addition, we measured physiological changes using heart rate variability before and after treatments, which was not investigated in previous studies.

## Methods

### Participants

A total of 61 participants who were diagnosed with panic disorder by a psychiatrist according to diagnostic criteria in the *Diagnostic and Statistical Manual of Mental Disorders, Fifth Edition*, at a psychiatric outpatient clinic (Yonsei University Gangnam Severance Hospital, Seoul, South Korea) were recruited for this study. To be included, participants met the diagnostic criteria for panic disorder (with or without agoraphobia) based on the Mini-International Neuropsychiatric Interview, had no change in drug dosage during the study period, and were aged 19 to 60 years. Patients with a history of major neurological or significant medical illness or who met the diagnostic criteria of current substance misuse were excluded. Written informed consent was acquired from all participants at the first visit. The study design and protocol were approved by the institutional review board of Yonsei University Gangnam Severance hospital (3-2018-0292). The trial was registered at ClinicalTrials.gov [NCT04985019].

### Procedure

#### Study Design

After participants were informed of the purpose of the study and consented to participate, for baseline assessment, a psychiatric interview by a psychiatrist or clinical psychologist was conducted and a self-rating questionnaire completed. A total of 54 participants were randomly assigned to either the VR treatment group or waitlist group after completion of the baseline assessment. Considering the dropout rates in previous VR studies were relatively high, we assigned patients to VR groups and waitlist groups in a ratio of 3:2 [40] in order to minimize the imbalance of group size. Randomization was carried out using R (version 4.0.2, R Foundation for Statistical Computing) with block sizes of 5. The allocation sequence was stratified for gender. Patients were naturally aware of the allocation. But the trained psychologist assessing clinician-administered scales was not able to know the patient’s group information from the beginning to the end of the study.

**Figure 1:**
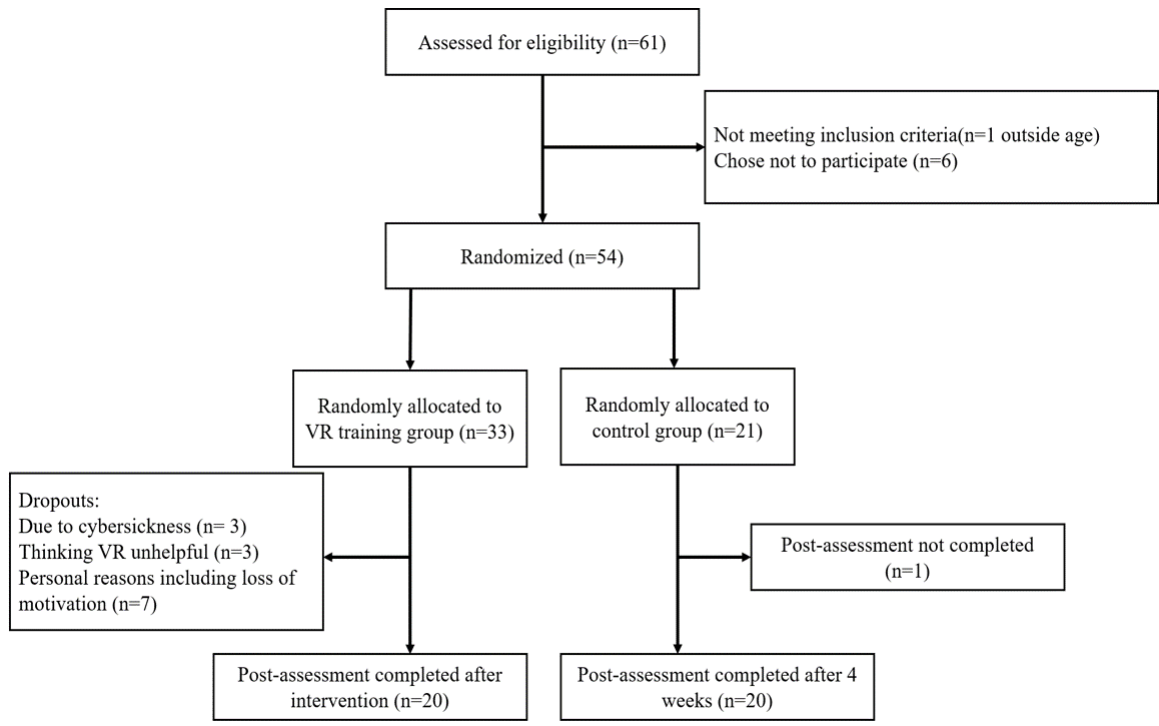
Flowchart of study process.

Participants assigned to the VR group were asked to complete the 4-week VR-based CBT for panic disorder following the guideline. Before the start of treatment, they received the VR devices with a use description. After the treatment, participants were asked to complete questionnaires on VR presence and stimulator sickness. After 4 weeks, participants were given the same measures as pretreatment except the diagnostic interview. Participants in the waitlist group went through the 2 assessment sessions within 4 weeks without any other treatment except the medication between assessments. Figure 1 presents a flowchart of this study.

#### VR Treatment

The VR treatment aimed at learning how to cope with panic symptoms and exposed participants to feared situations in a gradual and planned manner. The mobile app was designed to be used without a supporting therapist, although it can be used alongside a therapist or in a clinical setting if needed. The treatment plan for 4 weeks was designed to be 3 times per week, for a total of 12 sessions. Each session was 15 to 30 minutes depending on the module. All participants completed at least 12 sessions, and we allowed them to do more sessions or repeat modules if they wanted within the 4 weeks.

The VR system was developed on a mobile-based platform. The content was designed on Unity 2018.3.11f1 software (Unity Technologies). The avatars and structures comprising the virtual environment were built using a 3Ds Max 2014 (Autodesk). The program was installed on a Galaxy 8+ (Samsung Electronics) smartphone for use with a Gear VR (Samsung Electronics). All in-app data were collected on the smartphone with basic demographic information (eg, birth date and name). Video recordings of actors were taken with an Insta360 Pro camera (Insta360) to create a video-based virtual environment. Because most VR contents were based on video recordings, interactions with environments were limited in VR. However, users chose difficulty levels and training sessions they wanted among various contents and exposure stimuli in VR, indicating that the VR was a partially user-driven program.

**Figure 2:**
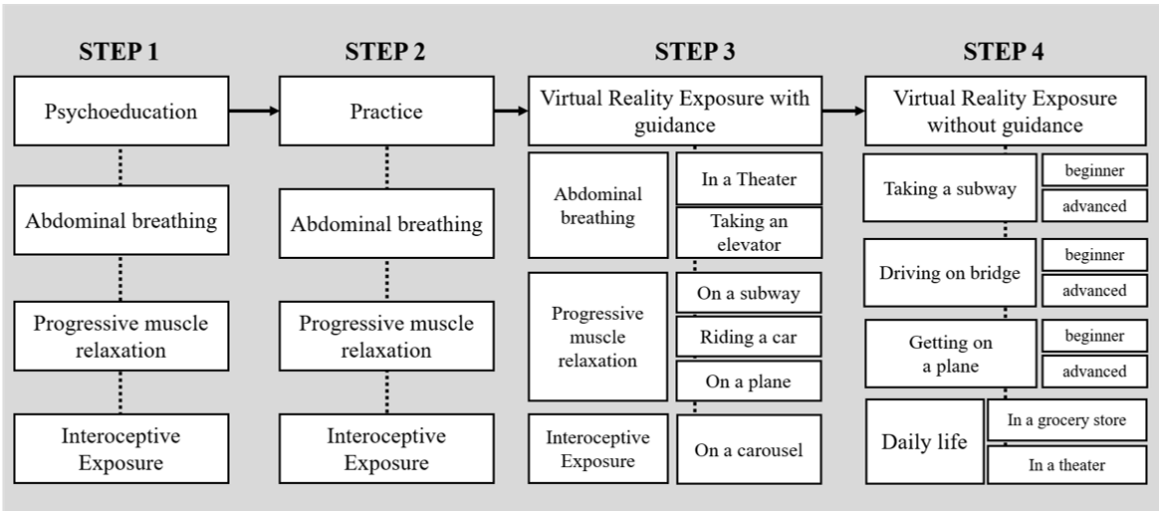
The content design of a virtual reality treatment application with 4 cognitive behavioral therapy components: psychoeducation, practice, virtual reality exposure with guidance, and virtual reality exposure without guidance.

**Figure 3:**
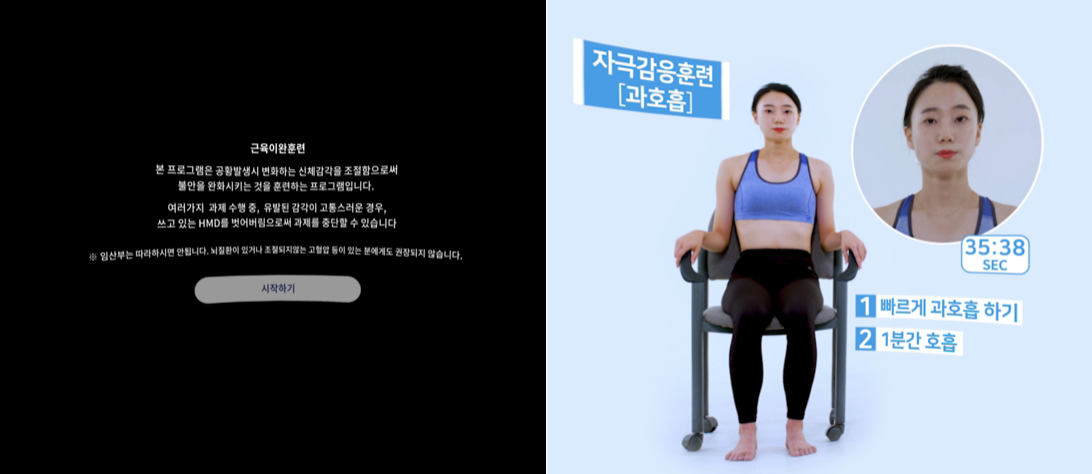
Virtual reality in the psychoeducation step: introduction to progressive muscle relaxation (left) and demonstration of interoceptive exposure training (right).

**Figure 4:**
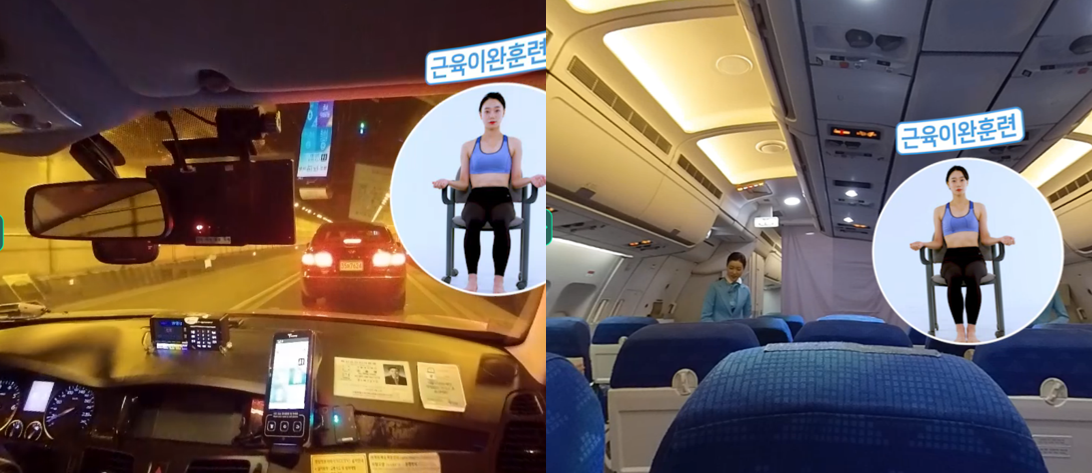
Virtual reality in the exposure with guidance step: driving in a tunnel (left) and taking a seat on a plane with the guidance of muscle relaxation (right).

**Figure 5:**
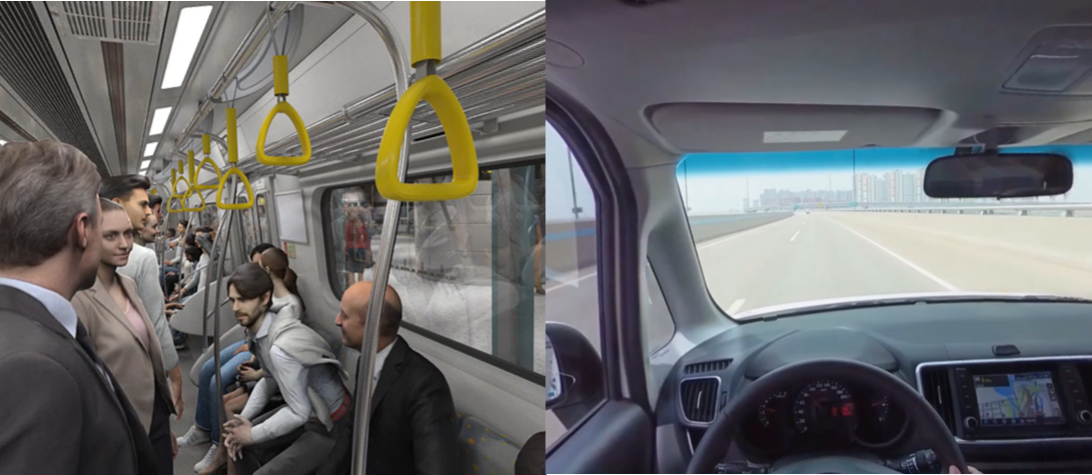
Virtual reality in the virtual reality exposure without guidance step: taking a subway with animated avatars (left) and driving on a bridge without guidance (right).

The VR consisted of 4 steps in which patients were gradually exposed to phobic stimuli while learning to cope with panic symptoms in each stage (Figure 2). The first was the psychoeducation step in which the patients learn how to breathe, relax, and be exposed to interoceptive stimuli and the reasons why these exercises reduce panic symptoms; they practiced what they learned in the practice step (Figure 3). Next were the two types of exposure therapy using VR in which participants experienced an immersive virtual environment comprising digital human avatars or video recordings of real people. In the exposure with guidance step, patients were exposed to various situations (eg, driving a car, taking an elevator) while using the skills they learned in previous steps under the virtual therapist’s guidance (the actor’s motion and the therapist’s voice; Figure 4). For example, while a participant was exposed to the driving a car scenario, a virtual therapist encouraged the patient to use abdominal breathing with a model’s demonstration and direct explanation. Each location had 2 or 3 levels of exposure manipulated by crowded density (eg, taking an elevator) or the time required to complete (eg, driving a car, getting on a plane or subway). After that, without a guide, for more than 5 minutes a patient was exposed to the situations that patients with agoraphobia are afraid of (eg, getting on a plane, driving on bridge; Figure 5). In the last step, 4 scenarios were divided into 2 levels, beginner and advanced, according to the difficulty such as length of time. After each VR session, participants completed questionnaires assessing their feeling of presence and their cybersickness symptoms.

#### Clinical Assessment

Clinical measures administered by trained psychologists at baseline and 4 weeks included the Panic Disorder Severity Scale (PDSS) [41], which consists of 7 items, each rated on a 5-point scale, assessing panic frequency, distress during panic, panic-focused anticipatory anxiety, phobic avoidance of situations, phobic avoidance of physical sensations, impairment in work functioning, and impairment in social functioning. The Hamilton Rating Scale for Depression (HRSD) [42], a depression assessment scale containing 17 items pertaining to symptoms of depression experienced over the past week, was also administered. Individual IQ scores were estimated using the short form of the Wechsler Adult Intelligence Scale–Revised [43].

Several measurements were conducted to assess the psychological state of participants. The Body Sensations Questionnaire (BSQ) [40] is a 17-item 5-point Likert scale that evaluates how afraid and anxious a patient is when they feel the physical sensation of each item in an unstable state. The Albany Panic and Phobia Questionnaire (APPQ) [44] is a 27-item 9-point Likert scale that was designed to measure the distinct dimension of fear in various situations with 3 subscales: social phobia, agoraphobia, and interoceptive fear. The Anxiety Sensitivity Index (ASI) [40] is a 16-item measure tapping the fear of anxiety sensations, known to be a risk factor for the development of panic. The State-Trait Anxiety Inventory (STAI) [45] has 20 items for assessing trait anxiety and 20 for state anxiety. The Hospital Anxiety and Depression Scale (HADS) [46] has 14 items with anxiety and depression subscales. The Korean Inventory of Social Avoidance and Distress Scale (K-SADS) [47] is a 28-item self-rated instrument used to measure various aspects of social anxiety including distress, discomfort, fear, anxiety, and avoidance of social situations. The Korean Inventory for Depressive Symptomatology (KIDS-SR) [48] is a self-report questionnaire that comprises symptoms of depression including melancholic, atypical, and anxious symptoms. The Perceived Stress Scale (PSS) [49] is a measure of the degree to which situations in one’s life are appraised as stressful.

After exposure to VR treatment, the 16-item Simulator Sickness Questionnaire (SSQ) [50] was used to assess participants’ subjective discomfort (disorientation, oculomotor symptoms, and nausea) to measure simulator sickness due to discrepancies between vision and motion after VR use.

#### Physiological Recording

Heart rate variability measurements of both groups were taken at baseline and after 4 weeks. Heart rate variability was assessed for 5 minutes in the frequency domain, recommended when examining autonomic nervous system activities [51], by using a SA-3000P arterial testing device (Medicore Co Ltd). During the measurement of heart rate variability, the participant stayed in the seated position without any movement. Electrodes on both wrists and the left ankle were used in this measurement procedure. The frequency domain measures were calculated as absolute and normalized powers of the power spectrum density in the high frequency (HF: 0.15 to 0.40 Hz) and low frequency (LF: 0.04 to 0.15 Hz) bands and LF/HF ratio. The HF bands are a marker of the parasympathetic tone, and LF bands correlate to sympathetic tone or to autonomic balance. LF/HF ratio is an index of the interaction between sympathetic and vagal activity [51].

### Statistical Analysis

To compare the difference between groups existing at baseline in terms of demographic and clinical variables, independent *t* tests for continuous variables and chi-square analyses for categorical variables were conducted. To assess the effects of the intervention on the clinical scales, we used analysis of covariance (ANCOVA) with randomization group as the independent variable and postscores for each variable as the dependent variables, controlling for baseline values of each outcome. Analyses on the mean differences between the two groups and confidence intervals were conducted to compare the two groups for each outcome. Effect sizes (Cohen *d*) divided by baseline pooled standard deviation were calculated for within- and between-group changes, with 0.2, 0.5, and 0.8 corresponding to small, medium, and large effect sizes [51]. We conducted completer analysis and intention-to-treat (ITT) analysis using multiple imputation with multivariate imputation by chained equations [52]. To examine the association between the use of VR and changes in the PDSS score, we conducted correlation analysis using the Pearson method. All statistical analyses were completed with R studio (R version 4.0.2, R Foundation for Statistical Computing). In all cases, a 2-tailed *P*<.05 was considered statistically significant. In addition, linear regression analysis was conducted to observe changes in SSQ scores over time, and Pearson correlation analysis was conducted to see the relationship between changes in SSQ and changes in clinical symptoms, including anxiety.

## Results

### Demographical and Clinical Characteristics

A total of 61 patients were screened for eligibility. Of these patients, 7 were excluded (see Figure 1) and 14 patients dropped out during the study (13 patients in the VR group and 1 patient in the waitlist group) because of various reasons; most of them did not complete the treatment according to the guideline. Three participants thought VR would not be helpful at the beginning of use, another 3 felt motion sickness while using VR, and the remaining 7 reported discontinuing due to personal reasons including loss of motivation. One participant in the waitlist group was absent at the follow-up assessment.

**Figure 6:**
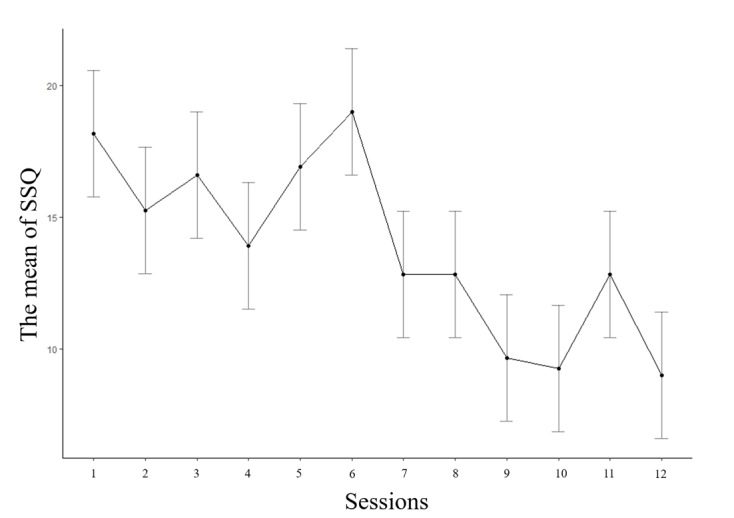
Mean Simulator Sickness Questionnaire scores for each session over time (bars shown: standard error of the mean, n=20).

At baseline, the 2 groups did not differ significantly regarding demographic characteristics or initial clinical assessment scores (Table 1). Demographic information of completers was described in Table 2, and there were no significant differences except for the PDSS scores. The average PDSS score in 13 dropout patients in the VR group was 12.46; the average in patients who did not drop out was 14.8 in the VR group and 11.95 in the waitlist group. The average of use time acquired from in-app data was 245 minutes (SD 61.44, median 250, range 105-457). Patients completed the SSQ every session, and the average SSQ from all patients and sessions was 12.26 (SD 13.26, median 6, range 0-48). There was no relationship between the SSQ scores and any clinical scales at both baseline and final assessment. Figure 6 displays change in SSQ over time. The number of sessions performed was significantly associated with lower SSQ scores (β=–.17, t_19_=–2.24, *P*=.03); however, there was no correlation between changes in SSQ and changes in any clinical scale we measured.

**Table 1:** Demographic and clinical characteristics at baseline by group.

Characteristics				VR^a^ training group (n=33)			Waitlist group (n=21)		t/χ^2^			*P* value
**Gender, n (%)**												
	Male		13 (39)			7 (33)		0.02			.87	
	Female		21 (60)			14 (67)		0.05			.82	
**Education, n (%)**												
	<High		10 (30)			12 (57)		2.84			.09	
	>College		23 (70)			9 (43)		—^b^			—	
**Occupation, n (%)**												
	Employed		18 (54)			13 (60)		0.23			.87	
	Unemployed		15 (45)			8 (40)		—			—	
Age (years), mean (SD)				35.84 (10.37)			37.14 (13.54)		0.30			.77
Duration of illness (months), mean (SD)				67.74 (65.70)			56.94 (66.34)		–0.25			.80
Intelligence score, mean (SD)				95.92 (17.40)			92.44 (11.01)		0.77			.44
**Psychotropic medication use**												
	Antidepressants, n (%)		30 (91)			19 (91)		0			>.99	
	Anxiolytics, n (%)		23 (70)			16 (76)		0.04			.83	
	HRSD^c^, mean (SD)		15.06 (8.85)			14.29 (5.87)		0.39			.70	
	PDSS^d^, mean (SD)		13.88 (4.29)			12.29 (3.35)		1.52			.13	
	**STAI^e^_TOTAL, mean (SD)**		55.36 (12.54)			53.43 (10.29)		0.01			.98	
		STAI_S^f^	53.58 (13.03)		55.43 (8.24)			0.62		.54		
		STAI_T^g^	108.94 (24.75)		108.86 (17.32)			–0.64		.52		
	KIDS-SR^h^, mean (SD)		15.55 (8.8)			17.43 (7.86)		–0.82			.42	
	PSS^i^, mean (SD)		19.97 (4.51)			19.05 (4.01)		0.78			.43	
	K-SADS^j^, mean (SD)		82.36 (21.24)			91.38 (19.23)		–1.61			.11	
	ASI^k^, mean (SD)		69.91 (32.88)			72.1 (28.55)		–0.26			.80	
	**HADS^l^, mean (SD)**		21.24 (9.12)			21.24 (7.08)		—			—	
		ANX^m^	11.27 (4.62)		11.38 (3.71)			–0.09		.92		
		DEP^n^	9.97 (4.83)		9.86 (3.64)			0.10		.92		
	**APPQ^o^, mean (SD)**		85.15 (43.86)			86.86 (48.82)		–0.13			.90	
		AGORA^p^	31.42 (14.78)		29.29 (20.25)			0.41		.67		
		SOCIAL^q^	27.52 (19.14)		36.19 (19.21)			–1.62		.11		
		INTERO^r^	26.21 (14.71)		21.38 (15.37)			1.14		.26		
	BSQ^s^, mean (SD)		58.03 (13.46)			59.71 (13.24)		–0.45			.65	

^a^VR: virtual reality.

^b^Not applicable.

^c^HRSD: Hamilton Rating Scale for Depression.

^d^PDSS: Panic Disorder Severity Scale.

^e^STAI: State and Trait Anxiety questionnaire.

^f^STAI_S: state anxiety.

^g^STAI_T: trait anxiety.

^h^KIDS-SR: Korean Inventory of Depressive Symptomatology.

^i^PSS: Perceived Stress Scale.

^j^K-SADS: Korean Inventory of Social Avoidance and Distress Scale.

^k^ASI: Anxiety Sensitivity Index.

^l^HADS: Hospital Anxiety and Depression Scale.

^m^ANX: Anxiety subscale of HADS.

^n^DEP: depression subscale of HADS.

^o^APPQ: Albany Panic and Phobia Questionnaire.

^p^AGORA: agoraphobia subscale of APPQ.

^q^SOCIAL: social anxiety subscale of APPQ.

^r^NTERO: interoceptive fear subscale of APPQ.

^s^BSQ: Body Sensations Questionnaire.

**Table 2:** Demographic and clinical characteristics of completers by group.

Characteristics				VR^a^ training group (n=20)		Waitlist group (n=20)			t/χ^2^			*P* value
**Gender, n (%)**				—		—			0.06			.80
	Male			8 (40)		7 (35)		—			—	
	Female			12 (60)		13 (65)		—			—	
**Education, n (%)**				—		—			1.63			.20
	<High			6 (30)		11 (55)		—			—	
	>College			14 (70)		9 (45)		—			—	
**Occupation, n (%)**				—		—			0			>.99
	Employed			12 (60)		12 (60)		—			—	
	Unemployed			8 (40)		8 (40)		—			—	
Age (years), mean (SD)				35.84 (10.37)		37.14 (13.54)			0.15			.87
Duration of illness (months), mean (SD)				64.25 (54.60)		44.10 (58.16)			1.12			.26
Intelligence score, mean (SD)				95.90 (12.47)		92.44 (11.01)			0.92			.35
**Psychotropic medication use**												
	Antidepressants, n (%)			19 (95)		18 (90)		0			>.99	
	Anxiolytics, n (%)			14 (70)		15 (75)		0			>.99	
	HRSD^b^, mean (SD)			14.15 (8.72)		14.20 (6.01)		–0.02			.98	
	PDSS^c^, mean (SD)			14.80 (4.18)		11.95 (3.05)		2.46			.01	
	**STAI^d^_TOTAL, mean (SD)**			108.30 (22.68)		109.35 (17.62)		0.20			.84	
		STAI_S^e^	54.35 (11.31)		53.65 (10.51)		–0.53			.59		
		STAI_T^f^	53.95 (12.02)		55.70 (8.35)		–0.16			.87		
	KIDS-SR^g^, mean (SD)			15.60 (8.47)		17.60 (8.02)		–7.66			.44	
	PSS^h^, mean (SD)			19.85 (4.52)		19.05 (4.11)		0.58			.56	
	K-SADS^i^, mean (SD)			84.45 (21.00)		92.80 (18.57)		–1.33			.19	
	ASI^j^, mean (SD)			73.85 (34.60)		74.65 (26.71)		–0.08			.93	
	**HADS^k^, mean (SD)**											
		ANX^l^	12.15 (4.25)		12.15 (4.25)		0.51			.60		
		DEP^m^	11.05 (4.21)		11.05 (4.21)		1.03			.30		
	**APPQ^n^, mean (SD)**			87.05 (45.15)		90.75 (46.63)		–0.25			.80	
		AGORA^o^	33.10 (14.27)		30.45 (20.04)		0.48			.63		
		SOCIAL^p^	27.65 (20.85)		37.85 (18.09)		–1.65			.10		
		INTERO^q^	26.30 (15.21)		22.45 (14.95)		0.80			.42		
	BSQ^r^, mean (SD)			60.55 (12.22)		61.40 (11.03)		–0.23			.81	

^a^VR: virtual reality.

^b^HRSD: Hamilton Rating Scale for Depression.

^c^PDSS: Panic Disorder Severity Scale.

^d^STAI: State and Trait Anxiety questionnaire.

^e^STAI_S: state anxiety.

^f^STAI_T: trait anxiety.

^g^KIDS-SR: Korean Inventory of Depressive Symptomatology.

^h^PSS: Perceived Stress Scale.

^i^K-SADS: Korean Inventory of Social Avoidance and Distress Scale.

^j^ASI: Anxiety Sensitivity Index.

^k^HADS: Hospital Anxiety and Depression Scale.

^l^ANX: Anxiety subscale of HADS.

^m^DEP: depression subscale of HADS.

^n^APPQ: Albany Panic and Phobia Questionnaire.

^o^AGORA: agoraphobia subscale of APPQ.

^p^SOCIAL: social anxiety subscale of APPQ.

^q^NTERO: interoceptive fear subscale of APPQ.

^r^BSQ: Body Sensations Questionnaire.

### ITT Analysis

#### Clinical Assessments

We repeated all tests using multiple imputation. Univariate ANCOVAs on the HRSD, PDSS, state anxiety (STAI_S), and PSS posttreatment scores, controlling for pretreatment scores (Multimedia Appendix 1), showed that the VR treatment group had significantly lower posttreatment scores than the control group for the HRSD (F_1,49_=5.96, *P*=.02, 
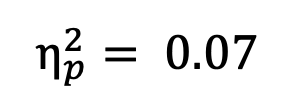
), PDSS (F_1,50_=9.20, *P*=.003, 
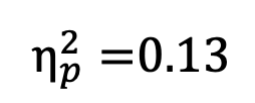
), STAI_S (F_1,50_=4.45 *P*=.04, 
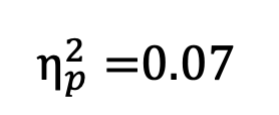
), and PSS (F_1,49_=4.56, *P*=.03, 
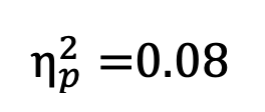
). Within-group effect sizes for the outcome measures are indicated in Multimedia Appendix 1. Large within-group effect sizes were found in the VR treatment group for the PDSS (*d*=1.05) and STAI_S (*d*=0.91). Moderate within-group effect sizes were found for the HRSD (*d*=0.68), STAI (*d*=0.65), anxiety subscale of HADS (*d*=0.59), and BSQ (*d*=0.57). Between-group differences of change were found in the PDSS (*P*<.01), STAI_S (*P*=.04), and PSS (*P*=.04).

#### Heart Rate Variability Frequency Domain Analysis

No significant difference was found at baseline in the mean values of total absolute power, normalized power, or LF/HF ratio between the two groups. As shown in Multimedia Appendix 2, only for the VR group, the mean normalized HF (nHF) power and LF/HF ratio were significantly increased after the VR treatment with moderate within-group effect sizes (LF/HF, *d*=0.53; nHF, *d*=0.52). But in the waitlist group, significant change and between-group difference of change were not found.

### Completer Analysis

#### Clinical Assessments

Univariate ANCOVAs on the PDSS and HADS scores, controlling for pretreatment scores (Table 2), showed that the VR treatment group had significantly lower posttreatment scores than the control group for PDSS (F_1,36_=6.16, *P*=.02, 
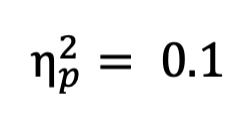
) and HADS (F_1,36_=4.66, *P*=.04, 
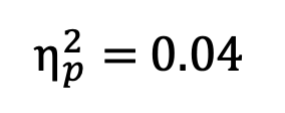
). Within-group effect sizes for the outcome measures are shown in Multimedia Appendix 3. Large within-group effect sizes were found in the VR treatment group for the PDSS (*d*=1.05) and STAI (*d*=0.98), and moderate within-group effect sizes were found for the HADS (*d*=0.69) and depression subscale of HADS (*d*=0.68). Between-group differences of change were found in the PDSS (*P*=.02) and HADS (*P*=.04).

#### Heart Rate Variability Frequency Domain Analysis

No significant difference was found at baseline in the mean values of total absolute power, normalized power, or the LF/HF ratio between the two groups. As shown in Multimedia Appendix 4, only for the VR group, the mean nHF power level was significantly increased after the treatment with moderate within-group effect sizes (nHF, *d*=0.55). In the waitlist group, significant change was not found. Between-group difference of change was found in the absolute HF power (*P*=.04).

## Discussion

### Principal Findings

This study aimed to investigate the effectiveness of mobile app-based VR for panic disorder in a randomized controlled design. To our knowledge, this is the first study to demonstrate the effectiveness of self-help VR to treat patients with panic disorder, while including not only the exposure technique but most other components of CBT such as psychoeducation, abdominal breathing, and progressive muscle relaxation. Our study demonstrated that self-help VR was effective for reducing panic disorder symptoms as assessed by the PDSS, compared with the waitlist group. These findings are inconsistent with previous meta-analyses of therapist-led VR for anxiety [53,54].

Although a group difference in clinical symptoms was not found at baseline, the comparison between completers showed a difference in PDSS score. The difference may be attributed to the dropout patients. This suggests that only those with severe symptoms remained to the end, and as the result, the average initial PDSS score in the VR group was increased. The severity of panic disorder symptoms might have been a motivation for participants to continue and to complete intervention, while the dropout patients with relatively mild symptoms discontinued use. In other words, our system might have been particularly useful for patients with severe symptoms. Considering the most common reason for dropout in VR exposure treatment is the failure to immerse in the VR environment [55], more severe panic disorder symptoms were probably able to elicit more anxiety in the VR environment, which might have increased the effectiveness of the treatment. In addition, given that fear of exposure is another common reason patients withdraw from exposure treatment [55], gradual and systematic exposure with coping techniques in our VR-based CBT program may have encouraged patients with more severe symptoms to continue the treatment. Our dropout pattern related to the severity and motivation can cause self-selection bias when analyzing only the completers, so we also examined ITT results.

We also observed a reduction in the STAI, suggesting that the VR group had a decrease in overall anxiety after 4 weeks of the VR program. Considering the significant difference between the two groups on HADS total score and adjusted mean changes of the VR group, VR treatment seemed to improve psychological distress. The full-scale HADS score has been used as a global measure of distress in several studies [56,57]. On the other hand, there was no significant change in other anxiety and depression scales including the HRSD and KIDS-SR among completers. Given that the program was designed to treat panic and agoraphobia symptoms, the effect may be limited to only such symptoms.

Contrary to our expectation, VR did not exhibit any significant influence on the APPQ and BSQ, which are thought to be highly related to panic disorder symptoms. The PDSS scale is a global assessment including panic attack frequency, distress during panic attacks, and work and social impairment, while the APPQ evaluates the fear of interoceptive sensation and agoraphobia. The 4-week study period might have been insufficient to reduce the specific fear itself. Otherwise, considering ITT analysis, it can be due to the reduced statistical power [58]. The ITT analysis using multiple imputation generally further confirmed that VR treatment groups experienced significant improvements in panic symptoms, anxiety, and depression including significant changes in the HRSD and BSQ.

Our positive results on psychological assessments including decreases in PDSS score were in line with our findings on heart rate variability data. The within-group results showed an increase in HF power and a decrease in LF/HF in only the VR group. Associations between panic disorder and low HF power, high LF power, and elevated LF/HF have been observed in numerous studies [59-63]. In particular, HF power is considered to be related to the parasympathetic activity [64]. Our results suggest there may be an improvement of the balance in the autonomic nervous system of the VR group participants; in other words, overactivation of sympathetic nervous system seems to be normalized after the treatment.

Previous studies showed that physiological symptoms of anxiety and cybersickness can overlap [65,66]. However, in the correlation analysis between the SSQ and clinical scales, we could not find a significant relationship although we observed a decrease in SSQ scores over time. There was a limitation that we did not measure clinical symptoms as frequently as the SSQ. However, at the very least this study revealed that global improvements in clinical symptoms were not due to patients becoming familiar with the VR environments.

Until recently, despite advantages of VR, most therapists have shown little interest in applying VR in their clinical practice [67]. One concern when applying VR to therapy is technical difficulties [68]. In this study, many participants completed the whole treatment process although they had never used VR devices, indicating the decent usability of VR. If patients are familiar with smartphones, they can easily overcome some inconvenience in mobile-based VR, and no additional training is required. In addition, this study demonstrated that mobile-based VR can be used by patients alone and exhibit positive results. It seems obvious that minimizing therapist effort is related to the cost effectiveness of the treatment [69].

In the context of minimizing therapist intervention, we used a virtual therapist who delivered psychoeducational contents and provided guided exposure to encourage the use of CBT techniques. Our result showed the possibility that patients eligible for VR could benefit from the immersive learning experience as well as exposure to feared stimuli under the guidance of a virtual therapist. Although its efficacy compared to the VR CBT without the presence of a virtual therapist remains to be investigated, a virtual therapist seems to be an essential component for fully self-guided VR treatment.

Additionally, it would be meaningful to try VR protocols including relaxation and breathing techniques and examine the therapeutic effects in future study. Based on recent studies describing the mechanisms of exposure therapy as inhibitory learning, the exposure to feared stimuli without coping skills may maximize the mismatch between expectations and experience and may enhance the learning effect rather than the exposure with coping skills [70]. Although traditional CBT protocol including coping skills have been used in this study, our VR protocol can be flexibly modified, so the recent protocol without coping skills can be applied as well. We could then conclude whether it shows better therapeutic effects.

### Limitations

There are several limitations of this study. First, our dropout rate was higher than a previous VR study [55] and similar to self-help treatment approaches [71-73]. As we noted, during the study periods, VR was fully executed by patients, so there was no other way to increase the treatment adherence of patients except for patient’s motivation. This lack of encouragement could be a limitation of a fully self-help VR approach, and in this study, it caused the initial difference in the clinical variable with high dropout rate. However, we tried to overcome this limitation by using ANCOVA and by also presenting the ITT analysis results. Second, due to the relatively small sample size, it is difficult to generalize the results even though we tried to provide the more objective physiological evidence as well [74]. It will be necessary to replicate our findings in larger samples. Third, given that we did not perform a follow-up assessment, long-term treatment effects remain unknown. Fourth, heart rate variability was measured only at rest. We would have been more informed if we had also measured heart rate variability in anxiety-provoking states. Finally, it is still not clear whether self-help VR is better than other treatment options since patients without any intervention were set as the waitlist group in this study.

### Conclusions

In summary, our findings support the hypothesis that self-guided, mobile app-based VR can reduce panic symptoms and help restore the autonomic nervous system. This study also suggested the decent feasibility of self-help VR for panic disorder, demonstrating the validity of the use of this new technique in real-world treatment. Future studies with larger sample size, longer duration of follow-up, and comparison with other treatment options will be able to verify and expand our results.

## Appendix

Multimedia Appendix 1 Changes in clinical variables at baseline and 4 weeks (intention-to-treat analysis).

Multimedia Appendix 2 Estimated mean and standard error of heart rate variability items (intention-to-treat analysis).

Multimedia Appendix 3 Changes of completers in clinical variables at baseline and 4 weeks (completer analysis).

Multimedia Appendix 4 Estimated mean and standard error of heart rate variability items (completer analysis).

Multimedia Appendix 5 CONSORT-eHEALTH checklist (V 1.6.1).

## Abbreviations

ANCOVA: analysis of covariance
APPQ: Albany Panic and Phobia Questionnaire
ASI: Anxiety Sensitivity Index
BSQ: Body Sensations Questionnaire
CBT: cognitive behavioral therapy
HADS: Hospital Anxiety and Depression Scale
HF: high frequency
HRSD: Hamilton Rating Scale for Depression
ITT: intention-to-treat
KIDS-SR: Korean Inventory of Depressive Symptomatology
K-SADS: Korean Inventory of Social Avoidance and Distress Scale
LF: low frequency
nHF: normalized high frequency
PDSS: Panic Disorder Severity Scale
PSS: Perceived Stress Scale
SSQ: Simulator Sickness Questionnaire
STAI: State-Trait Anxiety Inventory
STAI_S: state anxiety
VR: virtual reality
