# Supplementary material for: Effectiveness of Self-Guided Virtual Reality–Based Cognitive Behavioral Therapy for Panic Disorder: Randomized Controlled Trial
Source: JMIR Ment Health. 2021 Nov 22;8(11):e30590. doi: 10.2196/30590 (PMC8663599; doi:10.2196/30590)
Supplement: Multimedia Appendix 1 [file mental_v8i11e30590_app1.docx]

Multimedia Appendix 1. Changes in clinical variables at baseline and 4 weeks (ITT analysis).

|  | VR treatment within-group  mean change over time (4 weeks) | | | | waitlist within-group  mean change over time (4 weeks) | | | Between-group  Mean differences | | |
| --- | --- | --- | --- | --- | --- | --- | --- | --- | --- | --- |
|  | Adjusted mean change^q^  (SE)^s^ | 95% CI^t^ | Cohen's d^u^ | | Adjusted mean change  (SE) | 95% CI | Cohen's d | Adjusted mean difference | 95% CI | p value |
| **HRSD^b^** | -5.04(1.82) | -8.74 to -1.34* | | 0.68 | -1.21(1.75) | -4.86 to 2.44 | 0.21 | -3.83(1.95) | -0.23 to 7.89 | 0.02* |
| **PDSS^c^** | -4.2(0.99) | -6.21 to -2.19** | | 1.05 | -1.1(1.08) | -3.35 to 1.15 | 0.32 | -3.1(1.09) | 0.83 to 5.37 | <0.01** |
| **STAI^d^** | -10.2(2.77) | -24.27 to -3.13* | | 0.65 | -4.4(3.02) | -10.7 to 1.9 | 0.4 | -5.8(2.97) | -4.74 to 18.74 | 0.13 |
| **STAI_S^e^** | -4.3(2.81) | -15.85 to -4.55* | | 0.91 | -2.5(2.76) | -8.27 to 3.27 | 0.45 | -1.8(3.09) | -0.4 to 12 | 0.04* |
| **STAI_T^f^** | -13.7(5.19) | -10.03 to 1.43 | | 0.38 | -6.7(5.23) | -17.62 to 4.22 | 0.28 | -7(5.63) | -4.69 to 7.89 | 0.44 |
| **KIDS_SR^g^** | -0.9(1.75) | -4.46 to 2.66 | | 0.13 | -0.5(2) | -4.67 to 3.67 | 0.08 | -0.4(1.85) | -3.45 to 4.25 | 0.62 |
| **PSS^h^** | -1.7(1.05) | -3.85 to 0.45 | | 0.4 | 0.8(1.24) | -1.78 to 3.38 | 0.2 | -2.5(1.17) | 0.05 to 4.95 | 0.04* |
| **KSAD^i^** | -4.7(4.76) | -14.4 to 5 | | 0.24 | -1.2(5.67) | -13.04 to 10.64 | 0.07 | -3.5(5.32) | -7.59 to 14.59 | 0.43 |
| **ASI^j^** | -3.8(7.3) | -18.67 to 11.07 | | 0.13 | -2.4(8.2) | -19.52 to 14.72 | 0.09 | -1.4(7.83) | -14.93 to 17.73 | 0.80 |
| **HADS^k^** | -3.4(1.86) | -7.19 to 0.39 | | 0.45 | -1(1.93) | -5.03 to 3.03 | 0.16 | -2.4(1.97) | -1.7 to 6.5 | 0.13 |
| **ANX^l^** | -2.35(0.99) | -4.37 to -0.33* | | 0.59 | -0.62(1.06) | -2.82 to 1.58 | 0.18 | -1.73(1.05) | -0.47 to 3.93 | 0.06 |
| **DEP^m^** | -1.68(1.03) | -3.77 to 0.41 | | 0.4 | -0.43(1.05) | -2.63 to 1.77 | 0.13 | -1.25(1.09) | -1.03 to 3.53 | 0.18 |
| **APPQ^n^** | -2.4(8.92) | -20.57 to 15.77 | | 0.07 | 5.8(12.12) | -19.48 to 31.08 | 0.15 | -8.2(9.58) | -11.77 to 28.17 | 0.26 |
| **AGORA^o^** | -1(3.33) | -7.78 to 5.78 | | 0.07 | 0.7(5.15) | -10.05 to 11.45 | 0.04 | -1.7(3.69) | -6.01 to 9.41 | 0.63 |
| **SOCIAL^p^** | -3.6(3.98) | -11.7 to 4.5 | | 0.22 | 0.9(5.03) | -9.6 to 11.4 | 0.06 | -4.5(4.35) | -4.56 to 13.56 | 0.13 |
| **INTERO^q^** | 0.7(3.51) | -6.45 to 7.85 | | 0.05 | 3.6(4.42) | -5.62 to 12.82 | 0.25 | -2.9(3.85) | -5.14 to 10.94 | 0.44 |
| **BSQ^r^** | -8(3.44) | -15.01 to -0.99* | | 0.57 | -1.9(4.28) | -10.83 to 7.03 | 0.14 | -6.1(3.93) | -2.11 to 14.31 | 0.13 |

^a^Data are n (%) or mean ± standard deviation.

^b^HRSD, Hamilton Rating Scale for Depression;

^c^PDSS, Panic Disorder Severity Scale;

^d^STAI, the State and Trait Anxiety questionnaire;

^e^STAI_S, the state anxiety,

^f^STAI_T, the trait anxiety;

^g^KIDS_SR, Korean Inventory of Depressive Symptomatology;

^h^PSS, Perceived Stress Scale

^j^KSAD, Korean Inventory of Social Avoidance and Distress Scale

^j^ASI, Anxiety Sensitivity Index

^k^HADS, Hospital Anxiety and Depression Scale;

^l^ANX, Anxiety subscale of HADS;

^m^DEP, depression subscale of HADS

^n^APPQ, Albany Panic and Phobia Questionnaire;

^o^AGORA, agoraphobia subscale of APPQ;

^p^SOCIAL, social anxiety subscale of APPQ;

^q^INTERO, interoceptive fear subscale of APPQ;

^r^BSQ, Body Sensations Questionnaire;

^s^Adjusted mean change, Results from analysis of covariance models controlling for baseline values of criterion outcomes and psychotropic medication use (Mean_post_-Mean_baseline_)

^t^CI, confidence interval;

^u^Cohen’s d, (Mean_post_-Mean_baseline_)/ SD_diff_ with 0.2, 0.5, and 0.8 corresponding to small, medium, and large effect sizes, respectively;

*p < 0.05**p<0.01
