# Supplementary material for: Effectiveness of Self-Guided Virtual Reality–Based Cognitive Behavioral Therapy for Panic Disorder: Randomized Controlled Trial
Source: JMIR Ment Health. 2021 Nov 22;8(11):e30590. doi: 10.2196/30590 (PMC8663599; doi:10.2196/30590)
Supplement: Multimedia Appendix 2 [file mental_v8i11e30590_app2.docx]

Multimedia Appendix 2. Changes in heart rate variability items at baseline and 4 weeks (ITT analysis).

|  | VR treatment within-group  mean change over time (4 weeks) | | | waitlist within-group  mean change over time (4 weeks) | | | | Between-group  Mean differences | | |
| --- | --- | --- | --- | --- | --- | --- | --- | --- | --- | --- |
|  | Adjusted mean change(SE)^f^ | 95% CI^g^ | Cohen's d^h^ | Adjusted mean change(SE) | 95% CI | | Cohen's d | Adjusted mean change | 95% CI | p value |
| **HF(ms^2^)^a^** | 4.35(13.87) | -24.25 to 32.25 | 0.07 | 4 (15.89) | -29.14 to 37.14 | 0.08 | | -0.01(15.28) | -31.89 to 31.88 | 0.98 |
| **LF(ms^2^)^b^** | -3.8(6.33) | -16.69 to 9.09 | 0.15 | 2.5(9.42) | -17.17 to 22.17 | 0.08 | | 1.1(7.68) | -22.35 to 9.75 | 0.44 |
| **LF_HF^c^** | -0.06(0.03) | -0.12 to -0.003* | 0.53 | -0.01(0.04) | -0.1 to 0.08 | 0.07 | | -0.01(0.03) | -0.13 to 0.03 | 0.26 |
| **N_HF^d^** | 0.02(0.01) | 0.001 to 0.05* | 0.52 | 0.01(0.01) | -0.03 to 0.03 | 0.04 | | 0.002(0.01) | -0.01 to 0.05 | 0.20 |
| **N_LF^e^** | -0.01(0.01) | -0.04 to 0.01 | 0.29 | -0.01(0.02) | -0.04 to 0.03 | 0.04 | | 0.001(0.01) | -0.04 to 0.02 | 0.93 |

^a^LF, low frequency;

^b^HF, high frequency;

^c^LF_HF, Ratio LF [ms^2^]/HF[ms^2^];

^d^N_HF, HF power in normalized units HF/(total power-VLF)×100;

^e^N_LF, LF power in normalized units LF/(total power-VLF)×100;

^f^Adjusted mean change, Results from analysis of covariance models controlling for baseline values of criterion outcomes and psychotropic medication use (Mean_post_-Mean_baseline_)

^g^CI, confidence interval;

^h^Cohen’s d, (Mean_post_-Mean_baseline_)/ SD_diff_ with 0.2, 0.5, and 0.8 corresponding to small, medium, and large effect sizes, respectively;

*p < 0.05
