# Supplementary material for: Effectiveness of Self-Guided Virtual Reality–Based Cognitive Behavioral Therapy for Panic Disorder: Randomized Controlled Trial
Source: JMIR Ment Health. 2021 Nov 22;8(11):e30590. doi: 10.2196/30590 (PMC8663599; doi:10.2196/30590)
Supplement: Multimedia Appendix 3 [file mental_v8i11e30590_app3.docx]

Multimedia Appendix 3. Changes of completers in clinical variables at baseline and 4 weeks (Completer analysis).

|  | VR treatment within-group  mean change over time (4 weeks) | | | waitlist within-group  mean change over time (4 weeks) | | | Between-group  Mean differences | | |
| --- | --- | --- | --- | --- | --- | --- | --- | --- | --- |
|  | Adjusted mean  Change^s^  (SE) | 95% CI^t^ | Cohen's d^u^ | Adjusted  mean  change  (SE) | 95% CI | Cohen's d | Adjusted  mean  difference | 95% CI | p value |
| **HRSD^b^** | -3.1(2.12) | -7.53 to 1.33 | 0.46 | -1.1(1.58) | -4.4 to 2.2 | 0.22 | -2(2.12) | -2.43 to 6.43 | 0.1 |
| **PDSS^c^** | -4.05(1.22) | -6.6 to -1.5* | 1.05 | -1.42(1.07) | -3.65 to 0.81 | 0.42 | -2.63(1.24) | 0.03 to 5.23 | 0.02* |
| **STAI^d^** | -12(3.89) | -20.14 to -3.86* | 0.98 | -7(3.37) | -14.06 to 0.06 | 0.66 | -5(3.89) | -3.14 to 13.14 | 0.2 |
| **STAI_S^e^** | -7.8(5.27) | -18.84 to 3.24 | 0.47 | -4(4.19) | -12.78 to 4.78 | 0.3 | -3.8(5.27) | -7.24 to 14.84 | 0.07 |
| **STAI_T^f^** | -4.1 (3.02) | -10.43 to 2.23 | 0.43 | -2.7(2.88) | -8.72 to 3.32 | 0.3 | -1.4(3.02) | -4.93 to 7.73 | 0.57 |
| **KIDS_SR^g^** | -1.7(2.08) | -6.06 to 2.66 | 0.26 | -1.3(1.99) | -5.47 to 2.87 | 0.21 | -0.4(2.08) | -3.96 to 4.76 | 0.88 |
| **PSS^h^** | -1.2(1.29) | -3.89 to 1.49 | 0.3 | 0.9(1.22) | -1.64 to 3.44 | 0.23 | -2.1(1.29) | -0.59 to 4.79 | 0.07 |
| **KSAD^i^** | 0(5.29) | -11.07 to 11.07 | 0 | -1.4(4.81) | -11.47 to 8.67 | 0.09 | 1.4(5.29) | -12.47 to 9.67 | 0.69 |
| **ASI^j^** | -10.2(8.45) | -27.88 to 7.48 | 0.38 | -2.9(6.87) | -17.27 to 11.47 | 0.13 | -7.3(8.45) | -10.38 to 24.98 | 0.14 |
| **HADS^k^** | -4.7(2.15) | -9.21 to -0.19* | 0.69 | -1.3(1.97) | -5.43 to 2.83 | 0.21 | -3.4(2.15) | -1.11 to 7.91 | 0.04* |
| **ANX^l^** | -2.15(1.11) | -4.47 to 0.17 | 0.61 | -0.66(1.02) | -2.79 to 1.47 | 0.21 | -1.49(1.11) | -0.83 to 3.81 | 0.07 |
| **DEP^m^** | -2.51(1.17) | -4.95 to -0.07* | 0.68 | -0.63(1.08) | -2.88 to 1.62 | 0.19 | -1.88(9.82) | -18.66 to 22.42 | 0.06 |
| **APPQ^n^** | -8.6(11.46) | -32.58 to 15.38 | 0.24 | 6.3(11.75) | -18.3 to 30.9 | 0.17 | -14.9(11.46) | -9.08 to 38.88 | 0.06 |
| **AGORA^o^** | -5.3(3.86) | -13.39 to 2.79 | 0.43 | 0.7(4.98) | -9.73 to 11.13 | 0.04 | -6(3.86) | -2.09 to 14.09 | 0.07 |
| **SOCIAL^p^** | -4.8(5.09) | -15.45 to 5.85 | 0.3 | 0.9(4.54) | -8.6 to 10.4 | 0.06 | -5.7(5.1) | -4.97 to 16.37 | 0.05 |
| **INTERO^q^** | 0.5(4.12) | -8.12 to 9.12 | 0.04 | 4(4.07) | -4.52 to 12.52 | 0.31 | -3.5(4.12) | -5.12 to 12.12 | 0.29 |
| **BSQ^r^** | -5.7(3.28) | -12.56 to 1.16 | 0.55 | -2.1(3.06) | -8.5 to 4.3 | 0.22 | -3.6(3.28) | -3.26 to 10.46 | 0.17 |

^a^Data are n (%) or mean ± standard deviation.

^b^HRSD, Hamilton Rating Scale for Depression;

^c^PDSS, Panic Disorder Severity Scale;

^d^STAI, the State and Trait Anxiety questionnaire;

^e^STAI_S, the state anxiety,

^f^STAI_T, the trait anxiety;

^g^KIDS_SR, Korean Inventory of Depressive Symptomatology;

^h^PSS, Perceived Stress Scale

^j^KSAD, Korean Inventory of Social Avoidance and Distress Scale

^j^ASI, Anxiety Sensitivity Index

^k^HADS, Hospital Anxiety and Depression Scale;

^l^ANX, Anxiety subscale of HADS;

^m^DEP, depression subscale of HADS

^n^APPQ, Albany Panic and Phobia Questionnaire;

^o^AGORA, agoraphobia subscale of APPQ;

^p^SOCIAL, social anxiety subscale of APPQ;

^q^INTERO, interoceptive fear subscale of APPQ;

^r^BSQ, Body Sensations Questionnaire;

^s^Adjusted mean change, Results from analysis of covariance models controlling for baseline values of criterion outcomes and psychotropic medication use (Mean_post_-Mean_baseline_)

^t^CI, confidence interval;

^u^Cohen’s d, (Mean_post_-Mean_baseline_)/ SD_diff_ with 0.2, 0.5, and 0.8 corresponding to small, medium, and large effect sizes, respectively;

*p < 0.05**p<0.01
