# Supplementary material for: Effectiveness of Self-Guided Virtual Reality–Based Cognitive Behavioral Therapy for Panic Disorder: Randomized Controlled Trial
Source: JMIR Ment Health. 2021 Nov 22;8(11):e30590. doi: 10.2196/30590 (PMC8663599; doi:10.2196/30590)
Supplement: Multimedia Appendix 4 [file mental_v8i11e30590_app4.docx]

Multimedia Appendix 4. Changes in heart rate variability items at baseline and 4 weeks (Completer analysis).

|  | VR treatment within-group  mean change over time (4 weeks) | | | waitlist within-group  mean change over time (4 weeks) | | | | Between-group  Mean differences | | |
| --- | --- | --- | --- | --- | --- | --- | --- | --- | --- | --- |
|  | Adjusted mean change(SE)^f^ | 95% CI^g^ | Cohen's d^h^ | Adjusted mean change(SE) | 95% CI | | Cohen's d | Adjusted mean change | 95% CI | p value |
| **HF(ms^2^)^a^** | 7(15.77) | -26 to 40 | 0.14 | 8(15.51) | -24.47 to 40.47 | 0.16 | | 35(16.05) | 1.4 to 68.6 | 0.04* |
| **LF(ms^2^)^b^** | 1.3(8.03) | -15.5 to 18.1 | 0.05 | 0.5(9.17) | -18.69 to 19.69 | 0.02 | | 8.7(7.83) | -7.7 to 25.1 | 0.27 |
| **LF_HF^c^** | -0.04(0.03) | -0.11 to 0.03 | 0.39 | -0.03(0.04) | -0.11 to 0.04 | 0.28 | | -0.05(0.03) | -0.11 to 0.02 | 0.12 |
| **N_HF^d^** | 0.02(0.01) | 0.004 to 0.05* | 0.55 | 0.01(0.01) | -0.02 to 0.04 | 0.27 | | 0.02(0.01) | -0.01 to 0.05 | 0.11 |
| **N_LF^e^** | -0.02(0.01) | -0.05 to 0.01 | 0.46 | -0.01(0.01) | -0.04 to 0.02 | 0.27 | | -0.02(0.01) | -0.05 to 0.01 | 0.11 |

^a^LF, low frequency;

^b^HF, high frequency;

^c^LF_HF, Ratio LF [ms^2^]/HF[ms^2^];

^d^N_HF, HF power in normalized units HF/(total power-VLF)×100;

^e^N_LF, LF power in normalized units LF/(total power-VLF)×100;

^f^Adjusted mean change, Results from analysis of covariance models controlling for baseline values of criterion outcomes and psychotropic medication use (Mean_post_-Mean_baseline_)

^g^CI, confidence interval;

^h^Cohen’s d, (Mean_post_-Mean_baseline_)/ SD_diff_ with 0.2, 0.5, and 0.8 corresponding to small, medium, and large effect sizes, respectively;

*p < 0.05
